# Integrated exposure-based therapy for co-occurring post-traumatic stress and substance use among young people: a randomized controlled trial

**DOI:** 10.1080/20008066.2026.2691364

**Published:** 2026-07-21

**Authors:** Katherine L. Mills, Natalie Peach, Ivana Kihas, Katherine A. Dobinson, Joanne Cassar, Ashling Isik, Louise Bezzina, Olivia Schollar-Root, Vanessa E. Cobham, Emma L. Barrett, Sean Perrin, Sarah Bendall, Sudie E. Back, Kathleen Brady, Bronwyn Milne, Maree Teesson

**Affiliations:** a The Matilda Centre for Research in Mental Health and Substance Use, The University of Sydney, Sydney, Australia; b School of Psychology, University of Queensland, Brisbane, Australia; c Department of Psychology, Lund University, Sweden; d Orygen National Centre of Excellence in Youth Mental Health, Centre for Youth Mental Health, University of Melbourne, Parkville, Australia; e Department of Psychiatry and Behavioral Sciences, Medical University of South Carolina, Charleston, SC, USA; f Department of Adolescent Medicine, The Sydney Children’s Hospitals Network, Sydney, Australia

**Keywords:** Post-traumatic stress disorder, substance use disorder, adolescents and young adults, youth, symptom severity, randomized clinical trial, Trastorno de estrés postraumático, trastorno por uso de sustancias, adolescentes, adultos jóvenes, jóvenes, ensayo controlado aleatorizado

## Abstract

**Background:** Despite well-documented evidence demonstrating the efficacy and safety of integrated treatments for post-traumatic stress disorder (PTSD) and substance use disorder (SUD) in adults, few studies have been conducted among adolescents and young adults, developmental periods when these disorders typically have their onset.

**Objective:** This randomized controlled trial compared the efficacy of an integrated exposure-based treatment for PTSD and SUD among young people [Concurrent Treatment of PTSD and SUD Using Prolonged Exposure for Adolescents (COPE-A)] to a supportive counselling control condition [person-centred therapy (PCT)]. COPE-A represents an adaption of the evidence-based COPE treatment for adults, modified to meet the development needs of the target age group (12–25 years).

**Method:** Participants (*n* = 55; 69% female) were recruited in Sydney, Australia, between 2018 and 2022, and randomized to receive COPE-A or PCT. PTSD and substance use were assessed at study entry, and outcomes at 4 months (primary end-point) and 12 months post-baseline. Between-group differences in PTSD symptom severity (primary outcome), PTSD diagnosis, substance use, client satisfaction, and adverse events were examined.

**Results:** COPE-A showed significantly greater reductions in PTSD symptom severity [mean difference −9.82, 95% confidence interval (CI) −16.11 to −3.53] and PTSD diagnosis (odds ratio = 0.06, 95% CI 0.01 to 0.46) between baseline and 4 months, which were maintained through to 12 months. PCT demonstrated reductions in PTSD symptom severity, but these did not reach significance until 12 months (mean difference −8.92, 95% CI −13.41 to −4.43). Significant reductions in the frequency of substance use and severity of SUD were found between baseline and 12 months, but there were no between-group differences. Client satisfaction scores were significantly higher in COPE-A compared to PCT. There were no study-related adverse events.

**Conclusion:** The results provide evidence of the safety and efficacy of COPE-A in producing significantly greater improvements in PTSD symptom severity in a shorter time compared to PCT.

**Trial registration:** ACTRN12618000785202.

## HIGHLIGHTS

- This is the first study among adolescents and young adults to demonstrate the superiority of an integrated treatment for PTSD and SUD relative to a control condition in relation to PTSD outcomes.
- Accelerated reductions in PTSD symptom severity were observed among COPE-A (within 4 months) relative to PCT (12 months).
- COPE-A demonstrated higher rates of diagnostic remission at 4 month follow-up, with 20.0% of COPE-A meeting diagnostic criteria for PTSD compared to 66.7% of PCT.

## Abstract (es)

**Antecedentes:** A pesar de la evidencia bien documentada que demuestra la eficacia y seguridad de los tratamientos integrados para el trastorno de estrés postraumático (TEPT) y trastorno por uso de sustancias (TUS) en adultos, se han conducido pocos estudios en adolescentes y adultos jóvenes, los periodos del desarrollo en los que típicamente tienen su inicio.

**Objetivo:** Este ensayo controlado aleatorizado comparó la eficacia de un tratamiento integrado basado en la exposición para el TEPT y TUS en personas jóvenes (Tratamiento Concurrente de TEPT y TUS Utilizando Exposición Prolongada para Adolescentes: COPE-A) con una condición de control de terapia de apoyo (Terapia Centrada en la Persona; TCP). COPE-A representa una adaptación del tratamiento COPE para adultos basado en la evidencia, modificado para satisfacer las necesidades del desarrollo del grupo de edad objetivo (12–25 años).

**Método:** Se reclutaron participantes (*n* = 55; 69% mujeres) en Sídney, Australia entre el 2018 y 2022 y se les asignó aleatoriamente a recibir COPE-A o TCP. Se evaluaron el TEPT y el uso de sustancias al inicio del estudio y los resultados se evaluaron a los 4 meses (criterio de valoración principal) y a los12 meses después del inicio. Se evaluaron las diferencias entre grupos en la gravedad de los síntomas de TEPT (resultado primario), diagnóstico de TEPT, uso de sustancias, satisfacción del cliente y los eventos adversos.

**Resultados:** COPE-A mostró reducciones significativamente mayores en la gravedad de los síntomas de TEPT (diferencia media −9,82, IC 95%: −16.11 a −3,53) y el diagnóstico de TEPT (OR = 0,06, IC 95%: 0.01 a 0,46) entre el inicio y los 4 meses lo que se mantuvo hasta los 12 meses. La TCP mostró disminuciones en la gravedad de los síntomas de TEPT, pero estas no fueron significativas hasta los 12 meses (diferencia media −8,92, IC 95%: −13,41 a −4,43). Se encontraron reducciones significativas en la frecuencia del uso de sustancias y la gravedad del TUS entre el nivel basal y los 12 meses, pero no hubo diferencias entre grupos. Las puntuaciones de satisfacción del cliente fueron significativamente más altas en COPE-A en comparación con TCP. No hubo eventos adversos relacionados con el estudio.

**Conclusiones:** Los resultados proveen evidencia de la seguridad y eficacia del COPE-A en la producción de mejoras significativamente mayores en la gravedad de los síntomas de TEPT en un periodo más corto en comparación a la TCP.

## 1. Introduction

Research regarding the integrated treatment of post-traumatic stress disorder (PTSD) and substance use disorders (SUDs) has grown considerably over the past decade (Back et al., 2024; Roberts et al., 2022). PTSD and SUDs are complex interacting disorders that probably develop, and are maintained by, shared processes (e.g. avoidance-based mechanisms, shared neurological pathways) (Berenz et al., 2019) such that treating both in an integrated fashion produces synergistic therapeutic effects. Although there is no single pathway to the development of PTSD and SUD, the theory upon which integrated treatments are largely predicated is the self-medication hypothesis, which posits that SUDs develop as a consequence of the repeated use of substances to ameliorate PTSD symptoms (Hawn et al., 2020).

Several reviews and meta-analyses have concluded that trauma-focused therapies (those that involve exposure to, and processing of, memories, thoughts, feelings, and reminders of past trauma) delivered alongside treatment for substance use are effective in reducing PTSD symptom severity (Hien et al., 2023; Roberts et al., 2022). However, research to date has focused almost entirely on adults. Only three therapies have been developed for use with adolescents, only one of which is trauma-focused (Danielson et al., 2020; Fortuna et al., 2018; Najavits et al., 2006). A 2022 systematic review and meta-analysis of psychotherapies for co-occurring PTSD and SUD reported that the mean age of participants ranged from 33.7 to 54.0 years (> 50% reported a mean age > 40 years) across the 27 trials reviewed (Roberts et al., 2022). There is a clear need to develop and evaluate interventions that are developmentally appropriate to adolescents and young adults.

The imperative for studies to be conducted among adolescents and young adults lies in the potential for earlier intervention. Trauma exposure and the onset of these conditions peak in adolescence and young adulthood, with approximately 75% of SUDs and 50% of PTSD cases having their onset before the ages of 25–26 years (Barrett et al., 2026; Cabanis et al., 2021; Kelly et al., 2019). Trauma exposure during the formative developmental periods of childhood, adolescence, and young adulthood may have long-lasting effects not only on the stress response system, but also on neurotransmitter systems involved in the positive reinforcing effects of alcohol and other drugs, increasing vulnerability to the development of SUDs (Crews et al., 2007; De Bellis et al., 2005; Hinckley & Danielson, 2022; Hoffmann & Hoffmann, 2025; Moustafa et al., 2021). Young people with co-occurring PTSD and SUD have been found to exhibit significant internalizing and externalizing problems, including academic and vocational impairment, anxiety, depression, suicidality, poorer physical health, family and social dysfunction, aggression, and criminal behaviour (Cabanis et al., 2021; Darnell et al., 2019; Grummitt et al., 2024; Marusak et al., 2015; Rodriguez et al., 2019; Suarez et al., 2012). Long delays to accessing treatment for either disorder (median of 8 years for SUD, 7 years for PTSD) (Birrell et al., 2025), let alone integrated treatment, mean that by the time a person accesses treatment as an adult, a relationship of mutual maintenance has often been established, and the harms associated with the comorbidity have become entrenched. It is critical that interventions be provided earlier in the trajectory of these disorders, and for intervention effects to be demonstrated as soon as possible, to reduce the impacts of these disorders on neurological, cognitive, and social development, which may persist into adulthood.

This randomized clinical trial (RCT) examines the efficacy and safety of an integrated trauma-focused therapy adapted from an evidence-based adult treatment programme. Among adults, Concurrent Treatment of PTSD and Substance Use Disorders Using Prolonged Exposure (COPE) is the integrated treatment that has received the most empirical support to date (Back et al., 2024; Roberts et al., 2022) and the only integrated treatment recommended as a first-line treatment for co-occurring PTSD and SUD by the American Psychological Association (American Psychological Association, 2025). Five RCTs have examined the efficacy of COPE compared with a range of control conditions (including treatment as usual for SUD, relapse prevention, an active monitoring control condition, and a non-trauma-focused integrated treatment – Seeking Safety) in Australia (Mills et al., 2012), the USA (Back et al., 2019; Norman et al., 2019; Ruglass et al., 2017), and Sweden (Persson et al., 2025). COPE has consistently demonstrated superiority to control conditions in achieving greater reductions in PTSD symptom severity. Substance use outcomes have also significantly improved, but typically to the same degree as the control conditions. Importantly, there is no evidence of disproportionate worsening of substance use or clinical deterioration, which has historically been a concern among practitioners in relation to the treatment of PTSD among people with SUDs.

COPE-A represents an adaptation of the adult COPE manual tailored specifically to the needs of young people (Schollar-Root et al., 2022). This trial examines the efficacy and safety of COPE-A compared with person-centred therapy (PCT), a supportive counselling control condition. Findings are presented in relation to the primary outcome, change in PTSD symptom severity, and secondary outcomes relating to changes in PTSD diagnosis, substance use, client satisfaction, and adverse events. It was hypothesized that: (1) COPE-A would demonstrate greater pre-treatment (baseline) to post-treatment (4 month follow-up) reductions in PTSD symptom severity compared to PCT; (2) COPE-A would demonstrate reductions in substance use; and (3) changes observed from pre- to post-treatment would be maintained 12 months post-baseline.

## 2. Method

### 2.1. Trial design

A parallel-group, two-arm superiority trial using patient-level randomization was undertaken. The study protocol provides details on all aspects of the trial design (Mills et al., 2020), which was registered and updated with the Australia New Zealand Clinical Trials Registry (ACTRN12618000785202). The most significant update to the protocol was the expansion of the upper age limit to 25 years. At trial commencement it was intended that 100 adolescents aged 12–18 years would be recruited; however, recruitment was slower than anticipated [owing in part to the coronavirus disease 2019 (COVID-19) pandemic], necessitating this change. This upper age limit is, however, consistent with contemporary understandings of adolescence as a period that extends up to the age of 25 years (Baird et al., 2025).

### 2.2. Recruitment and eligibility criteria

**Figure 1.**
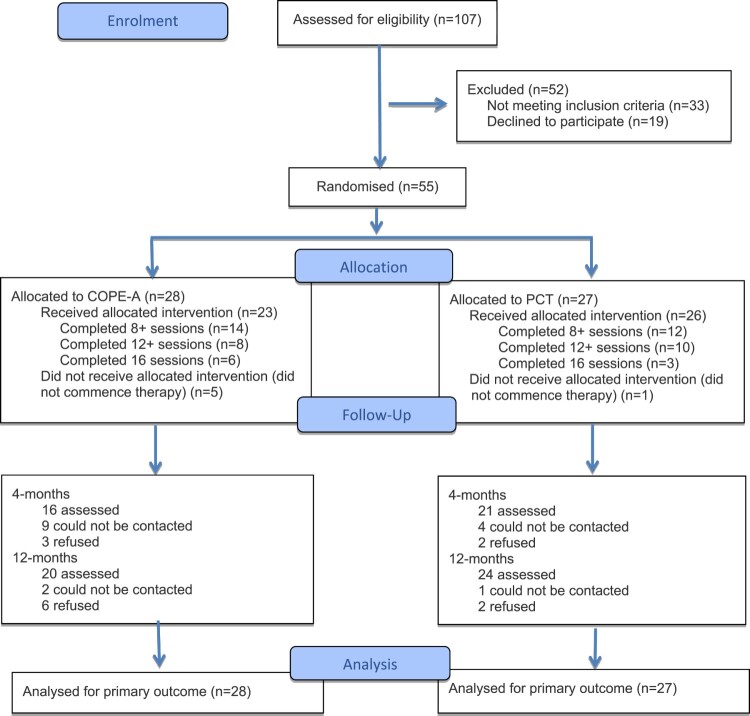
CONSORT flow diagram.

Fifty-five participants were recruited between June 2018 and June of 2022 from across Sydney via clinical services, community referrals, and targeted advertising, representing a 74.3% response rate (Figure 1). Inclusion criteria were: age 12–25 years; past-month substance use; score ≥ 2 on the CRAFFT (car, relax, alone, forget, friends, trouble) questionnaire (Knight et al., 1999), indicating a history of problematic substance use; exposure to at least one traumatic event [UCLA Post-Traumatic Stress Disorder Reaction Index (PTSD-RI)] (Pynoos & Steinberg, 2015); and a past-month Diagnostic and Statistical Manual of Mental Disorders, Fifth Edition (DSM-5) PTSD diagnosis or subthreshold PTSD (defined as meeting criterion A and at least one symptom across criteria B–G). Participants were excluded if they had a recent history of attempted suicide (i.e. within the past 3 months) or current risk (i.e. an active plan or intent) of suicide or serious self-harm; current symptoms of psychosis based on the Mini International Neuropsychiatric Interview for Children and Adolescents (score of > 1) (Sheehan et al., 2010) and clinical observation; cognitive impairment severe enough to impede treatment based on clinical observation; and ongoing trauma-related threat or ongoing unsupervised contact with the alleged perpetrator.

Written informed consent was obtained from all participants. Parent or caregiver consent was sought for those under the age of 16. If obtaining parental consent was not possible (e.g. parents unwilling to engage), adolescent consent alone was accepted for young people aged 14–15 years who were deemed to be mature minors. The study was approved by the Human Research Ethics Committees of the Sydney Children’s Hospital Network (HREC/17/SCHN/306) and the University of Sydney (2018/863).

### 2.3. Randomization

Participants were independently randomized to COPE-A or PCT (1:1 allocation ratio) following the baseline interview. To ensure balance between the groups, minimization was used with allocation according to gender (male vs female), age (12–14 vs 15–25 years), severity of SUD (mild: 2–3 criteria met, moderate: 4–5 criteria met, severe: ≥ 6 criteria met), trauma type (single incident vs multiple/prolonged incident), and initial PTSD symptom severity (subthreshold vs full PTSD criteria).

### 2.4. Interventions

Both arms offered up to 16, 60–90 min sessions with one of four registered psychologists employed on the trial. Trial therapists were trained in and delivered both therapies and received regular group supervision and support from a senior clinical supervisor. Both therapies included provision of an optional caregiver component (for parents, guardians, relatives, or other responsible adults from whom the young person may seek support), consisting of up to four 30 min sessions with the young person’s psychologist. The caregiver components provided psychoeducation about substance use and trauma responses, information about the treatment that the young person would undergo, and how the caregiver could best support the young person through therapy. With the exception of the first session, all caregiver sessions took place with the caregiver alone. In the first caregiver session, the participating young person was briefly present for discussion of the nature of caregiver’s involvement and confidentiality.

Sessions were audio recorded and reviewed weekly by the project coordinator using a checklist to document compliance. Fidelity was rated by a blind independent assessor for eight participants randomly selected from those who had commenced therapy (16.3% of those who commenced therapy; 14.6% of the total sample), stratified by therapist and treatment condition. In total, 36 sessions were rated. Average fidelity ratings were high, with a mean score of 4.88 (*SD* 0.35) out of a possible score of 5, indicating strong adherence to the treatment manuals.

#### 2.4.1. Concurrent Treatment of PTSD and SUD Using Prolonged Exposure – Adolescent version (COPE-A)

COPE-A is a developmentally adapted, integrated treatment targeting both PTSD and substance use, grounded in prolonged exposure and cognitive–behavioural therapy (CBT) approaches. Adapted from the evidence-based adult COPE protocol (Back et al., 2015), COPE-A incorporates core elements of trauma-focused CBT (including imaginal and in vivo exposure), CBT for substance use, psychoeducation, and motivational enhancement, with modifications for developmental stage (e.g. simplified rationales, age-relevant metaphors and examples, an enhanced focus on engagement, emotion regulation) (Schollar-Root et al., 2022).

#### 2.4.2. Person-centred therapy (PCT)

The comparison condition was manualized PCT (Kay-Lambkin et al., 2011), an active, non-directive intervention rooted in core principles of therapeutic change: genuineness, unconditional positive regard, and accurate empathy. Therapists provided empathic listening and support while participants were encouraged to lead the content of sessions, including when, how, and whether to discuss their trauma. PCT represents an approximation of current clinical practice with the added rigour of standardized delivery. PCT has been used among young people and shown to produce significant improvements in substance use and PTSD (Ehlers et al., 2010; Kay-Lambkin et al., 2011). To demonstrate superiority, COPE-A would need to show effects over and above those that can be attributed to the consequences of receiving attention from an interested person or the expectation of change.

### 2.5. Primary and secondary outcomes

Primary and secondary outcomes were assessed using structured interviews administered by research officers blind to treatment allocation. Interviews were conducted at baseline, and at 4 and 12 months post-baseline. Reimbursement was originally an A$40 voucher; this was increased to A$100 as participant burden increased following the start of the COVID-19 pandemic.

The primary outcome, PTSD symptom severity, was measured using the Clinician-Administered PTSD Scale for Children and Adolescents for DSM-5 (CAPS-CA-5) (Pynoos et al., n.d.). There are very limited data on the psychometric properties of the CAPS-CA-5 apart from studies that have validated versions translated from English to another language (Barroca et al., 2022; Kooij et al., 2025; Tanaka et al., 2024), but its predecessor (CAPS-CA) and the adult version (CAPS-5) upon which the CAPS-CA-5 is based have demonstrated acceptable psychometric properties (Wojujutari et al., 2024) and are considered the gold standard. Internal consistency in the present study was good for the total CAPS-CA-5 severity score (*α* = 0.84) and acceptable at the cluster level (B: *α* = 0.79; D: *α* = 0.68; E: *α* = 0.66).

The following secondary outcomes were also examined: PTSD symptom severity and diagnosis (using the CAPS-CA-5); frequency and quantity of substance use (timeline follow-back method) (Sobell & Sobell, 1992); severity of SUD [operationalized as number of DSM-5 criteria met, assessed using the Diagnostic Interview Schedule for Children, version 5 (DISC-5)] (Fisher, 2020). Substance use outcomes were examined in relation to participants’ main drug of concern. Standard alcohol units were calculated using Australian guidelines (National Health and Medical Research Council, 2020). Cannabis use was converted to estimated tetrahydrocannabinol (THC)-equivalent standard cannabis units following established guidelines (Freeman & Lorenzetti, 2020). Cannabis quantities were standardized across different delivery methods using published techniques (Dobson et al., 2026).

With the exception of the DISC, all instruments were administered in relation to the past month at baseline, and at 4 and 12 month follow-up. The DISC was administered in relation to past 12 months, and therefore only baseline and 12 month follow-up results are presented. Client satisfaction was assessed at the end of treatment by the project coordinator using the Youth Client Satisfaction Questionnaire (Shapiro et al., 1997).

### 2.6. Adverse events

An adverse event (AE) referred to an untoward occurrence during the trial, irrespective of whether it was causally related to the intervention or other aspects of trial participation. A serious adverse event (SAE) referred to any untoward occurrence that may have resulted in (or placed the participant in imminent risk of) death, serious injury/harm, severe or permanent disability, or unexpected/unplanned hospitalization; or where a participant’s actions placed someone else at imminent risk of serious harm. Potential AEs included arrest, going missing, unexpected homelessness, school suspension/expulsion/dropout, non-serious physical assault, and clinically significant deterioration of illness. Potential SAEs included exposure to life-threatening violence (victim/witness), sexual assault/abuse, suicide, serious self-harm, violent/ homicidal behaviour, hospitalization, and death. AEs were categorized as being either expected/anticipated or unexpected/unanticipated, study related or non-study related.

### 2.7. Data analysis

Missing data analysis revealed 18.5% missing data across the follow-up period, reflecting wave-level attrition. The results of Little’s missing completely at random (MCAR) test did not detect violations of the MCAR assumption (*χ*^2^_14_ = 13.48, *p* = .489). All available data were used in intention-to-treat analyses of outcomes using a series of generalized estimating equations (GEEs) for linear, binomial, and Poisson distributions. Robust standard errors were computed using the small-sample bias-corrected covariance estimator proposed by Mancl and DeRouen (2001). The end-point for the treatment outcome analyses was 4 months post-baseline (hypotheses 1 and 2); however, outcomes were examined through to 12 months post-baseline to determine the durability of effects (hypothesis 3). A sensitivity analysis was conducted for the primary outcome using multiple imputation to address missing data. Five imputed datasets were generated using the Markov chain Monte Carlo (MCMC) method, and results were pooled across imputations.

Individual quantity scores for participants’ main drug of concern were standardized (*z*-scores) to create a comparable composite quantity measure. This variable was transformed by adding 2 to remove negative values and log-transformed to correct for skewness. Between-group differences in time spent in treatment and client satisfaction were examined using Mann–Whitney U-tests. Fisher’s exact tests were used to examine between-group differences in AEs and SAEs. With the exception of the GEEs, which were modelled using R version 4.5.2, all analyses were conducted using IBM SPSS Statistics version 28.0.0.0. Differences were considered significant at *p* < .05.

## 3. Results

### 3.1. Sample recruitment and retention

Of the 107 people assessed for eligibility, 74 (71.2%) were eligible to participate (Figure 1). Primary reasons for exclusion were not meeting PTSD or substance use criteria (17.8%), risk or recency of suicide attempt/self-harm (5.6%), general instability/risk (6.5%), and unmanaged or severe psychosis (0.9%). Seventy-four per cent of those eligible agreed to participate. Thirty-eight (69.1%) were referred from clinical services; 16 (30.9%) were self-referrals made in response to advertising. Thirty-seven (67.3%) completed the 4 month follow-up and 44 (80.0%) completed the 12 month follow-up. Forty-six (83.6%) completed either follow-up; 35 (63.6%) completed both. Sample retention was not related to group allocation or sample characteristics.

### 3.2. Sample characteristics

The sample characteristics have been described by Peach et al. (2024). The mean age was 19.49 years (*SD* 3.27 years, range 13–25); 69.0% identified as female (Table 1). More than one-third had experienced a period of homelessness. Participants reported using a median of 4.0 (range 1–11) drug classes in the past month and 90.9% met diagnostic criteria for a severe SUD. Cannabis was the most commonly reported drug of choice (51.0% of participants), followed by alcohol (49.0%). The most common trauma types experienced were sexual assault; experiencing or witnessing physical abuse or threats of violence; death of a close loved one, and learning of violence towards a loved one that resulted in death or serious injury. All but one participant had experienced multiple or ongoing traumas (median of 6.0 trauma types, range 1–11) and 87.8% were diagnosed with PTSD, 38.2% with a dissociative subtype**.** No significant differences were observed between groups in relation to any of these variables.

**Table 1.** Demographic, substance use and trauma/post-traumatic stress disorder (PTSD)-related characteristics of the sample.

	COPE-A (*n* = 28)	PCT (*n* = 27)	Total (*n* = 55)
Demographics			
Age (years), mean (*SD*)	19.29 (3.32)	19.7 (3.26)	19.49 (3.27)
Identifying as female	19 (67.9)	19 (70.4)	38 (69.1)
Sexuality			
Heterosexual	17 (60.7)	14 (51.9)	31 (56.4)
Gay/lesbian/homosexual	1 (3.6)	3 (11.1)	4 (7.3)
Bisexual	6 (21.4)	8 (29.6)	14 (25.5)
Other	4 (14.3)	2 (7.4)	6 (10.9)
Australian born	26 (92.9)	25 (92.6)	51 (92.7)
Currently attending school or tertiary education	17 (60.7)	20 (74.1)	37 (67.3)
Ever suspended	16 (61.5)	11 (40.7)	27 (50.9)
Ever expelled	2 (7.7)	1 (3.7)	3 (5.7)
Ever arrested	10 (35.7)	6 (22.2)	16 (29.1)
Current living arrangements			
Parents’ home	11 (39.3)	12 (44.4)	23 (41.8)
Other family home	2 (7.1)	2 (7.4)	4 (7.3)
Own house or flat	12 (42.9)	13 (48.1)	25 (45.5)
Shelter/refuge	3 (10.7)	0 (0)	3 (5.5)
Ever homeless	13 (46.4)	8 (29.6)	21 (38.2)
Juvenile detention history	4 (14.3)	1 (3.7)	5 (9.1)
Prison history	1 (3.6)	0 (0)	1 (1.8)
Substance use	** **	** **	
Age at first use (years), mean (*SD*)	12.25 (3.01)	12.82 (3.05)	12.53 (3.02)
Drug of choice			
Cannabis	14 (50.0)	13 (48.1)	27 (49.1)
Alcohol	12 (42.9)	14 (51.9)	26 (47.3)
Ecstasy/non-prescribed amphetamines	2 (7.2)	0	2 (7.2)
No. of SUD criteria met, median (range)	9 (1–11)	9 (3–11)	9 (1–11)
Diagnosis of SUD	27 (96.4)	27 (100)	54 (98.2)
Severity of SUD (DISC)			
Mild or below	2 (7.2)	1 (3.7)	3 (5.4)
Moderate	1 (3.6)	1 (3.7)	2 (3.6)
Severe	25 (89.3)	25 (92.6)	50 (90.9)
No. of drug types used, median (range)^a^			
Past month	4 (1–9)	5 (2–11)	4 (1–11)
Lifetime	10 (5–17)	10 (2–15)	10 (2–17)
No. of days used any substance in past month, median (range)	20.5 (3–28)	18 (1–28)	20 (1–28)
Trauma/PTSD-related			
Event types			
Sexual molestation	21 (75.0)	18 (66.7)	39 (70.9)
Physical abuse at home – witnessed	18 (64.3)	18 (66.7)	36 (65.5)
Death of close loved one	19 (67.9)	15 (55.6)	34 (61.8)
Beaten up/shot at/threatened – experienced	20 (71.4)	13 (48.1)	33 (60.0)
Learn of violent death/serious injury of loved one	18 (64.3)	14 (51.9)	32 (58.2)
Physical abuse at home – experienced	15 (53.6)	16 (59.3)	31 (56.4)
Beaten up/shot at/threatened – witnessed	18 (64.3)	13 (48.1)	31 (56.4)
Sexual abuse – rape	16 (57.1)	13 (48.1)	29 (52.7)
Seen a dead body	10 (35.7)	6 (22.2)	16 (29.1)
Bad accident	6 (21.4)	5 (18.5)	11 (20.0)
Traumatic medical treatment	4 (14.3)	3 (11.1)	7 (12.7)
War	3 (10.7)	1 (3.7)	4 (7.3)
Natural disaster	3 (10.7)	1 (3.7)	4 (7.3)
Other traumatic events	9 (32.1)	13 (48.1)	22 (40.0)
Simple/single incident	0 (0)	1 (3.7)	1 (1.8)
Complex/multiple incident	28 (100)	26 (96.3)	54 (98.2)
No. of trauma types experienced, median (range)	6 (1–11)	5 (1–9)	6 (1–11)
Age of first trauma exposure (years), median (range)	6 (1–19)	5 (0–16)	5 (0–19)
Age at index trauma exposure (years), mean (*SD*)	12.14 (5.90)	10.44 (6.41)	11.31 (6.16)
PTSD diagnosis (CAPS-CA-5)			
Full diagnosis	25 (89.3)	22 (81.5)	47 (85.5)
Dissociative subtype	10 (35.7)	11 (40.7)	21 (38.2)
Delayed onset	6 (21.4)	9 (33.3)	15 (27.3)
PTSD symptom severity (CAPS-CA-5), mean (*SD*)			
Total	34.75 (10.49)	36.19 (9.91)	35.45 (10.14)
Re-experiencing	8.71 (3.97)	8.67 (3.49)	8.69 (3.71)
Avoidance	4.18 (1.44)	3.52 (1.53)	3.85 (1.51)
Negative cognitions and mood	13.00 (3.63)	14.04 (4.86)	13.51 (4.27)
Hyperarousal	8.86 (3.76)	9.96 (3.88)	9.40 (3.82)
Duration of symptoms (years), median (range)	3 (0.17–21)	2 (0.08–22)	3 (0.8–22)

Note: Data are shown as *n* (%) unless otherwise indicated.

COPE-A = Concurrent Treatment of PTSD and SUD Using Prolonged Exposure, Adolescent version; PCT = person-centred therapy; SUD = substance use disorder; DISC = Diagnostic Interview Schedule for Children; CAPS-CA-5 = Clinician-Administered PTSD Scale for Children and Adolescents for DSM-5.

^a^ Included alcohol, cannabis, tobacco, amphetamines (prescribed and non-prescribed, including methamphetamines), cocaine, ecstasy, LSD, heroin, other opioids (prescribed and non-prescribed), GHB, ketamine, kava, inhalants, steroids, synthetic cannabis, synthetic stimulants, and benzodiazepines (prescribed and non-prescribed).

### 3.3. Treatment attendance

There were no significant differences between groups in relation to treatment attendance. Twenty-four participants (85.7%) randomized to COPE-A and 25 participants (92.6%) randomized to PCT attended at least one session (i.e. started treatment). Among those randomized to COPE-A, the median number of sessions attended was 7 (range 0–16; median = 9 among those who started treatment). The median number of sessions attended by those randomized to PCT was 6.0 (range 0–16, median = 6.5 among those who started treatment). The percentage of COPE-A completing ≥ 8, ≥ 12, and 16 sessions was 50.0%, 28.6%, and 21.4%, respectively. The corresponding percentages for PCT were 44.4%, 37.0%, and 11.1%. Uptake of the caregiver component was low for both COPE-A (*n* = 3) and PCT (*n* = 5). The median number of caregiver sessions attended was 4 (range 1–4) and 3 (range 1–4) for the COPE-A and PCT groups, respectively.

### 3.4. Outcomes

Descriptive and inferential statistics for outcomes related to PTSD and substance use are provided in Tables 2 and 3, respectively.

**Table 2.** Within- and between-group change in post-traumatic stress disorder (PTSD) symptom severity and diagnosis.

Outcome measure	Mean (*SE*)			Mean difference					
CAPS scores	Baseline	4 months	12 months	Within-group difference between baseline and 4 months	Between-group difference between baseline and 4 months	Within-group difference between 4 and 12 months	Between- group difference between 4 and 12 months	Within-group difference between baseline and 12 months	Between- group difference between baseline and 12 months
Total severity score									
COPE-A	34.75 (2.02)	21.47 (3.37)	24.64 (3.31)	−13.28 (2.72)^a^	−9.82 (3.41)^b^	3.17 (3.42)	8.63 (4.12)^d^	−10.11 (3.11)^a^	−1.19 (3.92)
PCT	36.19 (1.94)	32.72 (2.84)	27.27 (2.66)	−3.46 (2.05)	Ref.	−5.46 (2.30)^d^	Ref.	−8.92 (2.39)^a^	Ref.
Between-group difference at each interview, mean difference (*SE*)	−1.44 (2.80)	−11.25 (4.04)^d^	−2.63 (4.25)						
PTSD diagnosis	% (*SE*)			OR					
COPE-A	89.3 (5.84)	20.0 (7.66)	31.6 (8.79)	0.03 (2.51)^a^	0.06 (2.84)^c^	2.03 (2.40)	4.56 (2.64)	0.05 (2.11)^a^	0.27 (2.64)
PCT	81.5 (7.47)	66.7 (9.07)	47.8 (9.61)	0.43 (1.64)	Ref.	0.44 (1.52)	Ref.	0.19 (1.86)^c^	Ref.
Between-group difference at each interview, OR (*SE*)	1.89 (2.26)	0.11 (2.43)^d^	0.52 (2.00)						

Note: COPE-A = Concurrent Treatment of PTSD and SUD Using Prolonged Exposure, Adolescent version; PCT = person-centred therapy; CAPS = Clinician-Administered PTSD Scale; OR = odds ratio; Ref. = reference value.

^a^ *p* ≤ .001; ^b^*p* < .005; ^c^*p* < .01; ^d^*p* < .05.

**Table 3.** Within- and between-group change in substance use outcomes.

Outcome measure	Mean			IRR					
Main drug of concern	Baseline	4 months	12 months	Within-group difference between baseline and 4 months	Between-group difference between baseline and 4 months	Within-group difference between 4 and 12 months	Between-group difference between 4 and 12 months	Within-group difference between baseline and 12 months	Between-group difference between baseline and 12 months
Frequency (number of days used)									
COPE-A	14.96 (1.89)	13.55 (2.29)	9.38 (2.10)	0.90 (0.84)	0.98 (1.37)	0.75 (1.17)^d^	0.98 (1.20)	0.63 (1.15)^d^	0.96 (1.20)
PCT	14.22 (1.88)	13.27 (3.07)	10.05 (2.45)	0.86 (1.33)	Ref.	0.76 (1.10)	Ref.	0.66 (1.35)	Ref.
Between-group difference at each interview, IRR (*SE*)	1.05 (1.21)	1.03 (1.24)	1.01 (1.20)						
Severity of SUD									
COPE-A	8.32 (0.45)	N/A	5.83 (0.66)	N/A	N/A	N/A	N/A	0.70 (1.12)	0.95 (1.00)
PCT	8.41 (0.39)	N/A	6.27 (0.71)	N/A	Ref.	N/A	Ref.	0.74 (1.13)^c^	Ref.
Between-group difference at each interview, IRR (*SE*)	0.99 (1.18)	N/A	0.94 (1.00)						
Standardized quantity (*z* scores)				Mean difference					
COPE-A	0.24 (0.31)	−0.15 (0.13)	−0.09 (0.17)	−0.11 (0.08)	−0.12 (0.10)	0.02 (0.03)	0.06 (0.09)	−0.08 (0.10)	−0.06 (0.11)
PCT	−0.01 (0.12)	0.00 (0.26)	−0.05 (0.16)	0.01 (0.07)	Ref.	−0.04 (0.09)	Ref.	−0.03 (0.07)	Ref.
Between-group difference at each interview, mean difference (*SE*)	0.04 (0.09)	−0.08 (0.10)	−0.01 (0.09)						

Note: COPE-A = Concurrent Treatment of PTSD and SUD Using Prolonged Exposure, Adolescent version; PCT = person-centred therapy; IRR = incidence rate ratio; Ref. = reference value.

^a^ *p* ≤ .001; ^b^*p* < .005; ^c^*p* < .01; ^d^*p* < .05.

#### 3.4.1. PTSD symptom severity and diagnosis

##### 3.4.1.1. Symptom severity

There was a significant group × time interaction in relation to PTSD symptom severity (*χ*^2^_2_ = 9.01, *p* = .011). COPE-A demonstrated significantly greater reductions in total CAPS scores compared to PCT between baseline and 4 months [mean difference −9.82, 95% confidence interval (CI) −16.49 to −3.14], such that there was a significant between-group difference in CAPS scores at 4 month follow-up (mean difference −11.25, 95% CI −19.88 to −2.62). The reductions observed in COPE-A were maintained through to 12 month follow-up. PCT did not demonstrate a significant reduction between baseline and 4 month follow-up, but a significant reduction was observed between baseline and the 12 month follow-up (mean difference −8.92, 95% CI −13.61 to −4.23). There was no significant between-group difference in scores at 12 months. The sensitivity analysis yielded results consistent with the primary analysis. Findings relating to change in individual PTSD symptoms clusters are presented in the Supplementary material.

##### 3.4.1.2. Diagnostic remission

There was a significant group × time interaction in relation to PTSD diagnostic remission (*χ*^2^_2_ = 7.29, *p* = .026). COPE-A demonstrated significantly greater reductions in rates of PTSD diagnosis compared to PCT between baseline and 4 months (OR = 0.06, 95% CI 0.01 to 0.46) such that there was a significant between-group difference in the rates of diagnostic remission at 4 month follow-up (COPE-A 20.0% vs PCT 66.7%; OR = 0.11, 95% CI 0.02 to 0.65). COPE-A demonstrated a non-significant increase in the rate of PTSD between 4 and 12 months but continued to demonstrate a significantly lower rate than that observed at baseline (COPE-A: 12 months 31.6% vs baseline 89.3%; OR = 0.05, 95% CI 0.01 to 0.23). PCT did not demonstrate a significant reduction between baseline (81.5%) and 4 month follow-up (66.7%), but a significant reduction was observed between baseline and the 12 month follow-up (47.8%; OR = 0.19, 95% CI: 0.06 to 0.65). There was no significant difference in rates of PTSD diagnosis between-groups at 12 months.

#### 3.4.2. Substance use

##### 3.4.2.1. Frequency

The group × time interaction in relation to the number of days that the main drug of concern was used in the past month was not significant, indicating that the direction and degree of change did not differ significantly between groups. When the interaction effect was removed from the model, a significant effect for time was found (*χ*^2^_2_ = 7.51, *p* = .023), indicating a significant reduction in the number of days that the main drug of concern was used in the past month between baseline and 12 months for the sample overall [incidence rate ratio (IRR) = 0.64, 95% CI 0.46 to 0.89]. No significant difference was found in relation to this outcome between baseline and 4 months, or between groups across time.

##### 3.4.2.2. Quantity

The group × time interaction in relation to the quantity of the main drug of concern used in the past month was not significant, indicating that the direction and degree of change did not differ significantly between groups. No significant main effects for time or group were observed after removal of the interaction term from the model, indicating no significant change in relation to this outcome between groups or across time.

##### 3.4.2.3. Severity of SUD

The group × time interaction in relation to the number of SUD criteria met for the main drug of concern in the past 12 months was not significant, indicating that the direction and degree of change did not differ significantly between groups. When the interaction effect was removed from the model, a significant effect for time was found (*χ*^2^_1_ = 16.52, *p* < .001), indicating a significant reduction in the number of dependence criteria met for the sample overall between baseline and 12 months (IRR = 0.72, 95% CI 0.62 to 0.84). No significant difference was found between baseline and 4 months, or between groups across time.

### 3.5. Client satisfaction

Twenty participants from each group completed the client satisfaction questionnaire. The COPE-A group reported significantly higher client satisfaction scores compared to the PCT group (COPE-A: median 49.5, range 24–55; PCT: median 46.0, range 17–53; *U* = 278, *p* = .035).

### 3.6. Adverse events

Forty-two AEs or SAEs were reported in relation to 12 participants (21.8%; eight COPE-A, four PCT). Ten participants experienced an AE (18.2%; seven COPE-A, three PCT) and five experienced an SAE (9.1%; three COPE-A, two PCT). The most common event types experienced were having been arrested (10.9%; three COPE-A, three PCT) and having been the victim of a physical assault (10.9%; four COPE-A, two PCT). Four were hospitalized (7.3%; two COPE-A, two PCT). Two participants (both PCT) attempted suicide and one (COPE-A) engaged in self-harm (COPE-A). All events were classified as expected for this patient population and no events were deemed to be study related. No age-related difference was observed between those who had experienced an AE or SAE and those who had not.

## 4. Discussion

The present study represents one of the first studies to examine the efficacy of an integrated treatment for traumatic stress and substance use among adolescents and young adults. Consistent with our hypotheses, and the findings of studies conducted among adults experiencing this comorbidity, participants randomized to receive COPE-A demonstrated significantly greater pre- to post-treatment reductions in PTSD symptom severity compared with those randomized to PCT, and these improvements were maintained through to the 12 month follow-up. A similar pattern was observed in relation to PTSD diagnosis, with 68–80% no longer meeting criteria for PTSD during the follow-up phase. As expected, those randomized to receive PCT also demonstrated reductions in PTSD diagnosis and symptom severity, but these reductions did not reach significance until the 12 month follow-up. These effects are likely to represent the benefits that can be attributed to factors common to all forms of successful psychotherapies: genuineness or congruence, unconditional positive regard, and accurate empathy. The superior effects observed in COPE-A indicate are over and above those that can be attributed to the consequences of receiving attention from an interested person or the expectation of change.

Although consistent with findings from studies relating to the treatment of comorbid SUD and PTSD among adults, this is the first study among adolescents and young adults to demonstrate the superiority of an integrated treatment relative to a control condition in relation to PTSD outcomes. In a larger RCT of the only other adolescent trauma-focused treatment available to date, Risk Reduction through Family Therapy (RRFT), a 24-session family-based, trauma narrative–focused intervention (Danielson et al., 2020), found that both those randomized to RRFT and those randomized to treatment as usual demonstrated comparable improvements in PTSD symptoms throughout the entirety of the study. As noted by the authors, however, trauma-focused CBT as a standard of care may have featured heavily in the treatment-as-usual control arm of the study. The only other integrated treatment that has been evaluated in an RCT, Seeking Safety (a 25 session present/non-trauma-focused treatment), also did not find a significant difference in PTSD outcomes compared with a treatment-as-usual control group (Najavits et al., 2006). Thus, the findings from this study provide evidence of the efficacy of COPE-A in producing significantly greater improvements in PTSD symptom severity not only in a shorter period of time compared to PCT, but also relative to other treatments evaluated to date. As COPE-A was not directly compared to RRFT or Seeking Safety in the present study, this conclusion can only be considered as tentative. The potential of these accelerated gains is nonetheless of great clinical import given the critical window of brain maturation in which they are occurring, and the benefits associated with accelerated gains on longer term outcomes (Aderka et al., 2011).

Regarding substance use outcomes, all interaction effects were non-significant, indicating comparable change across both groups. Modest reductions were observed between baseline and 12 months in relation to the frequency of substance use and severity of SUD, but no changes were observed in relation to the quantity used. These findings are contrary to those of Danielson et al. (2020), who found greater reductions in the number of days used among young people randomized to receive RRFT, but these differences did not emerge until the 12 month follow-up in their trial. The lack of between-groups differences, as well as the slower and more moderate response observed in relation to substance use outcomes compared with PTSD outcomes is, however, consistent with the adult literature (Hien et al., 2023; Roberts et al., 2022). Findings of a meta-analysis of treatment approaches for adults indicate that substance use outcomes may be optimized when trauma-focused treatments are accompanied by pharmacotherapies for substance use (Hien et al., 2023). Further research to examine this potential among adolescents and young people is needed.

High attrition is a common feature of most community-based trials of treatments for SUD and PTSD, irrespective of the type of intervention utilized (Roberts et al., 2022). Encouragingly, the majority of participants across both groups in the current study had commenced treatment; of those, the median number of sessions completed was nine for the COPE-A group and six for the PCT group. One in five participants in the COPE-A group completed all 16 sessions available to them, compared with one in 10 in the PCT group. Combined with higher client satisfaction scores among the COPE-A group, these findings indicate that COPE-A may be perceived more favourably by clients, a conclusion that is consistent with the broader literature on client preferences in PTSD treatment indicating a stronger preference for exposure and other evidence-based trauma-focused therapies (Simiola et al., 2015).

Although uptake of treatment was high among adolescents and young people participating in the trial, the same cannot be said for the optional caregiver component. This finding was mostly driven by participants not wishing to have a caregiver involved. This lack of caregiver involvement is in stark contrast to the study by Danielson et al. (2020), where caregiver participation was the norm. This difference in caregiver participation may also explain, at least in part, differences in young people’s session attendance, with Danielson et al. (2020) reporting a mean of 18.53 sessions for RRFT and 12.51 sessions for treatment as usual out of a possible of 24 sessions. There are, however, other methodological differences between these studies that may explain the difference in findings (e.g. sample size, characteristics, and eligibility criteria).

The findings from this study need also be considered within the context of the severe clinical profile (Peach et al., 2024) and high rate of AEs experienced by participants during the trial period, none of which was study related, and all were expected in the context of the population under investigation. It is notable that significant improvements in PTSD symptoms and substance use were able to be obtained in this context, and this demonstrates the resilience of the population under study. This study also extends prior research conducted among adults attesting to the safety of delivering integrated exposure-based treatments to adolescents and young people with SUDs, even in the presence of continued substance use, other comorbidities, and life adversity.

The findings from this study need to be considered in light of several limitations. The sample size was considerably smaller than anticipated (*n* = 100) and therefore the study was underpowered, meaning that only differences of larger magnitude could be detected. Although this was due in part to the impact of the COVID-19 pandemic, the slower than expected recruitment rate also reflects the challenge of recruiting complex samples of relatively high-risk young people to such trials. Recruitment challenges are characteristic of trials examining the efficacy of psychological interventions for young people aged ≤ 18 years, even in the absence of complex comorbidities, with a systematic review of 70 studies reporting an average sample size of 79 (Hoppen et al., 2025). Recruitment difficulties led to the expansion of the age range for this sample to include young adults, which may impact on the capacity to make comparisons with other trials of adolescent groups. It should be noted, however, that there were very few differences in the clinical characteristics of those within the 12–17-year-old age bracket compared with those aged 18–25 (Peach et al., 2024), a finding that is consistent with contemporary understandings of adolescence as a period that extends up to the age of 25 years (Baird et al., 2025). Overall, caution should be taken in generalizing the findings of such a small sample, which need to be replicated in larger studies, with greater representation from those in the adolescent age group.

Although the sample characteristics did not differ between the COPE-A and PCT groups, nor did they impact on sample retention (80% at 12 months), the findings may have been influenced by other confounding factors and loss to follow-up. Given the considerable literature demonstrating the reliability and validity of self-reported substance use, particularly in the context of clinical trials and situations where there are no adverse consequences of reporting use (Bharat et al., 2023), no secondary biomarkers were obtained. Nonetheless, the potential for recall or social desirability bias must be acknowledged. By focusing on participants’ primary substance of concern, changes to secondary substance use have also not been captured. These limitations underscore the need for larger, methodologically rigorous studies examining the efficacy of COPE-A as well as other treatment approaches for adolescents and young adults.

## 5. Conclusion

Despite the limitations of this study, the results provide evidence of the efficacy of an integrated exposure-based therapy in producing significantly greater improvements in PTSD symptom severity in a shorter time compared to PCT, and in fewer sessions relative to other treatments investigated to date. Although not examined in this study, the faster rate of improvement among those randomized to receive COPE-A may have flow-on effects, leading to more rapid improvements in other domains that have been impacted by a young person’s PTSD symptoms, such as social and occupational functioning. Empirical evidence for the safety of both approaches is also supported by a lack of study-related AEs, and improvements were achieved despite high rates of non-study-related AEs that are characteristic of the population under study.

## Supplementary Material

Online only supplementary material.docx

## Data Availability

Owing to ethical considerations and the sensitive nature of the research, supporting data are not available.
