# Supplementary material for: Integrated exposure-based therapy for co-occurring post-traumatic stress and substance use among young people: a randomized controlled trial
Source: Eur J Psychotraumatol. 2026 Jul 21;17(1):2691364. doi: 10.1080/20008066.2026.2691364 (PMC13390176; doi:10.1080/20008066.2026.2691364)
Supplement: Online only supplementary material.docx [file ZEPT_A_2691364_SM2335.docx]

**Change in the severity of PTSD symptoms clusters**

**Results**

Descriptive and inferential statistics for outcomes related to PTSD and substance use are provided in supplementary table 1.

Re-experiencing symptoms: There was a significant group × time interaction in relation to the severity of re-experiencing symptoms (χ^2^_2_ = 8.74, p = .001). COPE-A demonstrated a significant reduction in re-experiencing symptoms between baseline and 4-months (mean difference -2.71, 95%CI -4.77 to -0.64) which was maintained through to 12-month follow-up. PCT did not demonstrate a significant reduction between baseline and the 4-month follow-up, but did demonstrate a significantly greater reduction in re-experiencing symptoms between the 4-month and 12-month follow-up compared to COPE-A (mean difference --3.46, 95%CI: -1.12 to -5.79) such that there was no significant difference in between-group scores at 12-months.

Avoidance symptoms: There was a significant group × time interaction in relation to the severity of avoidance symptoms (χ^2^_2_ = 9.19, p = .019). COPE-A demonstrated significantly greater reductions in avoidance symptoms compared to PCT between baseline and 4-months (mean difference -2.19, 95%CI: -3.62 to -0.77) such that there was a significant between-group difference in avoidance scores at 4-month follow-up (mean difference -1.53, 95%CI: -2.96 to -0.10). The reductions observed in COPE-A were maintained through to 12-month follow-up. PCT did not demonstrate a significant reduction between baseline and the 4-month follow-up, but a significant reduction was observed between baseline and the 12-month follow-up (mean difference -1.11, 95%CI: -2.00 to -0.23). Overall COPE-A demonstrated a significantly greater reduction in avoidance symptoms compared to PCT between baseline and 12-months (mean difference = -1.37, 95%CI: -2.69 to -0.05).

Negative mood and cognitions: There was a significant group × time interaction in relation to the severity of negative mood and cognitions (χ^2^_2_ = 8.76, p = .012). COPE-A demonstrated significantly greater reductions in negative mood and cognitions compared to PCT between baseline and 4-months (mean difference -4.63, 95%CI: -7.70 to -1.55) such that there was a significant between-group difference in avoidance scores at 4-month follow-up (mean difference -5.66, 95%CI: -9.11 to -2.22). The reductions observed in COPE-A were maintained through to 12-month follow-up. PCT did not demonstrate a significant reduction between baseline and the 4-month follow-up, but a significant reduction was observed between baseline and the 12-month follow-up (mean difference -3.31, 95%CI: -5.50 to -1.11) such that there was no significant difference in between-group scores at 12-months.

Hyperarousal symptoms: The group × time interaction in relation to hyperarousal symptoms (χ^2^_2_ = 0.44, p = .80) was not significant indicating that the direction and degree of change in symptom severity did not differ between groups. No significant main effects for time or group were observed after removal of the interaction term from the model, indicating no significant change in relation to this outcome between groups or across time.

**Supplementary Table 1:** *Within- and between-group change in PTSD symptom cluster severity*

| **Outcome**  **measure** | **Mean (SE)** | | | **Mean difference** | | | | | |
| --- | --- | --- | --- | --- | --- | --- | --- | --- | --- |
| **CAPS scores** | Baseline | 4-months | 12-months | Within-group difference between baseline and 4-months | Between-group difference between baseline and 4-months | Within-group difference between 4- and 12-months | Between- group difference between 4- and 12-months | Within-group difference between baseline and 12-months | Between- group difference between baseline and 12-months |
| **Cluster B: Re-Experiencing** |  |  |  |  |  |  |  |  |  |
| COPE-A | 8.71 (0.76) | 6.01 (1.11) | 6.74 (0.94) | -2.71 (1.05)^d^ | -2.62 (1.33) | 0.73 (0.97) | 3.46 (1.19)^b^ | -1.97 (1.05) | 0.84 (1.26) |
| PCT | 8.67 (0.68) | 8.58 (0.92) | 5.85 (0.73) | -0.09 (0.82) | [Reference] | -2.72 (0.69)^a^ | [Reference] | -2.81 (0.70)^a^ | [Reference] |
| Between-group difference at each interview, mean difference (SE) | 0.05 (1.03) | -2.57 (1.44) | 0.89 (1.19) |  |  |  |  |  |  |
| **Cluster C: Avoidance** |  |  |  |  |  |  |  |  |  |
| COPE-A | 4.18 (0.28) | 1.92 (0.50) | 1.70 (0.45) | -2.26 (0.57)^a^ | -2.19 (0.73)^b^ | -0.22 (0.52) | 0.82 (0.64) | -2.48 (0.50)^a^ | -1.37 )0.68)^d^ |
| PCT | 3.52 (0.30) | 3.45 (0.53) | 2.41 (0.46) | -0.07 (0.45) | [Reference] | -1.04 (0.38)^b^ | [Reference] | -1.11 | [Reference] |
| Between-group difference at each interview, mean difference (SE) | 0.66 (0.41) | -1.53 (0.73)^d^ | -0.71 (0.64) |  |  |  |  |  |  |

**Supplementary Table 1 (continued):** *Within- and between-group change in PTSD symptom cluster severity*

| **Outcome**  **measure** | **Mean (SE)** | | | **Mean difference** | | | | | |
| --- | --- | --- | --- | --- | --- | --- | --- | --- | --- |
| **CAPS scores** | Baseline | 4-months | 12-months | Within-group difference between baseline and 4-months | Between-group difference between baseline and 4-months | Within-group difference between 4- and 12-months | Between- group difference between 4- and 12-months | Within-group difference between baseline and 12-months | Between- group difference between baseline and 12-months |
| **Cluster D: Negative alterations in cognition and mood** |  |  |  |  |  |  |  |  |  |
| COPE-A | 13.00 (0.70) | 6.76 (1.34) | 8.30 (1.45) | -6.24 (1.25)^a^ | -4.63 (1.57)^b^ | 1.54 (1.57) | 3.23 (1.95) | -4.70 (1.44)^b^ | -1.39 (1.82) |
| PCT | 14.04 (0.95) | 12.42 (1.14) | 10.73 (1.14) | -1.62 (0.96) | [Reference] | -1.69 (1.15) | [Reference] | -3.31 (1.12)^b^ | [Reference] |
| Between-group difference at each interview, mean difference (SE) | -1.04 (1.18) | -5.66 (1.76)^ab^ | -2.43 (1.85) |  |  |  |  |  |  |
| **Cluster E: Alterations in arousal and reactivity** |  |  |  |  |  |  |  |  |  |
| COPE-A | 8.86 (0.72) | 6.97 (1.15) | 7.94 (0.94) | -1.89 (0.96) | -0.31 (1.19) | 0.97 (1.20) | 0.94 (1.44) | -0.92 (0.89) | 0.63 (1.26) |
| PCT | 9.96 (0.76) | 8.39 (0.83) | 8.42 (0.93) | -1.58 (0.69)^d^ | [Reference] | 0.03 (0.80) | [Reference] | -1.55 (0.89) | [Reference] |
| Between-group difference at each interview, mean difference (SE) | -1.11 (1.05) | -1.42 (1.42) | -0.48 (1.32) |  |  |  |  |  |  |

**Discussion**

The pattern of change observed in relation to re-experiencing (criterion B) and negative cognitions and mood (criterion D) were similar to that observed for overall PTSD symptom severity. So too for avoidance (criterion C), with the exception being that the difference between groups was maintained through to 12-month follow-up. Between-group differences were not observed in relation to hyperarousal (criterion E), with scores remaining at levels similar to those at baseline for both groups across time. There is a notable gap in research relating to the impact of PTSD treatment in relation to individual symptom clusters among young people, but there is evidence among adults to suggest that this cluster may be more persistent than others^1^, and other adjunctive approaches (e.g., meditation-based approaches, pharmacotherapies) may be beneficial in addressing these symptoms^2^. The effective treatment of this symptom cluster is particularly important among young people given that it has been shown to play a unique and strong role in influencing the trajectory of other symptom clusters^3^, and may therefore impact on a person’s capacity to retain gains made in relation to other symptom clusters.

**References**

1. Schnurr PP, Lunney CA. Residual symptoms following prolonged exposure and present‐centered therapy for PTSD in female veterans and soldiers. *Depression and anxiety*. 2019;36(2):162-169.

2. Crawford JN, Talkovsky AM, Bormann JE, Lang AJ. Targeting hyperarousal: Mantram Repetition Program for PTSD in US veterans. *European Journal of Psychotraumatology*. 2019;10(1):1665768.

3. Schell TL, Marshall GN, Jaycox LH. All symptoms are not created equal: the prominent role of hyperarousal in the natural course of posttraumatic psychological distress. *Journal of abnormal psychology*. 2004;113(2):189.
